# Spinal Dural Arteriovenous Fistula and Cecal Arteriovenous Malformation in a Boy

**DOI:** 10.21699/ajcr.v8i1.510

**Published:** 2017-01-05

**Authors:** Vimlesh Soni, Pankaj C Vaidya, Jitendra Kumar Sahu, Mukesh Yadav, Pratibha Singhi

**Affiliations:** 1 Department of Paediatrics, Advanced Paediatrics Center, Postgraduate Institute of Medical Education and Research, Chandigarh, India; 2 Department of Radiodiagnosis, All India Institute of Medical Sciences, Delhi

**Keywords:** Dural - Arteriovenous fistula, Cecum - Arteriovenous malformation

## Abstract

Concurrent spinal dural arteriovenous fistula (AVF) and cecal arteriovenous malformation (AVM) are very rare. A 6-year old boy presented with lower limb paresis after trauma. On imaging work-up spinal dural AVF was found. It was managed with endovascular glue embolization. After two years, the boy presented with severe anemia and occult gastrointestinal tract (GIT) bleed. Cecal AVM was diagnosed and managed with embolization.

## INTRODUCTION

Spinal dural AVF are vascular malformations located in the dura mater of the spinal cord that result in arteriovenous shunting. Although rare, they account for approximately 80% of all spinal vascular abnormalities.[1] Vascular malformations of the gastrointestinal tract are also rare in children.[2] Concurrent spinal dural AVF and cecal AVM have not been reported till now.

## CASE REPORT

A 6-year-old boy presented with weakness of both lower limbs after trauma after fall from 8 feet height. On examination, he had power of 1/5 in both lower limbs with hyporeflexia and hypotonia. Bowel and bladder functions were also affected. MRI of spine was suggestive of abnormal flow voids in cervico-dorsal spine (C6-D4) with intramedullary bleed (Fig.1a,b). Digital subtraction angiography suggested dural arteriovenous fistula with arterial feeder from radiculopial branches of left D6 intercostal artery with venous drainage into multiple perimedullary veins (Fig. 2a). Endovascular glue embolization of dural AVF was done with 0.4 ml of N-butyl cyanoacrylate-lipiodol. Angiography post-embolization showed glue cast at fistula site and no opacification of arterial feeder (Fig. 2b). Child had gradual improvement in lower limb weakness on follow up.

Two years later, the child again presented with progressive pallor for one month duration. On examination, he had severe pallor and residual lower limb weakness with power of 2/5. Other systemic examination was normal. On investigation child had haemoglobin of 2.9 gm/dl. Peripheral smear showed microcytic hypochromic anemia. He was transfused with packed red cells. There was decline in hemoglobin even after blood transfusion. Further work up for anemia suggested stool positive for occult blood. As child already had AV malformation of one site, AV malformation of gut was also suspected. CT angiography of mesenteric vessels showed AVM of Iliocolic artery (Fig. 2c). Glue embolization of AVM was done under digital subtraction angiography (Fig. 2d). Check angiogram of intracranial vessels showed no abnormality. Child has normal hemoglobin after one year follow up.

**Figure:**
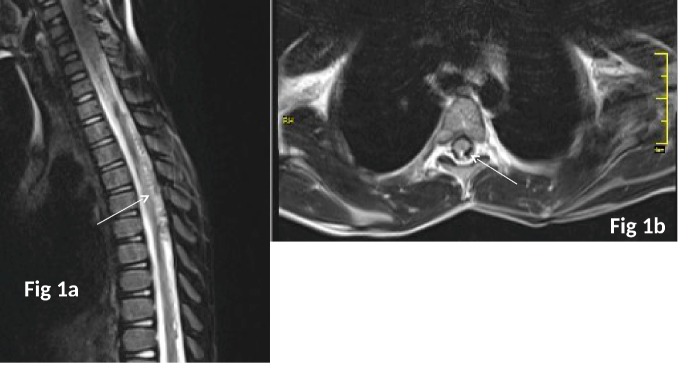
Figure 1: Sagittal T2 Weighted Images (Figure 1a) of Spine showing abnormal flow voids (white arrow) posterior to spinal cord in cervico-dorsal spine (C6-D4). MRI Spine (T2 Weighted axial Images- Figure 1b) showing abnormal flow voids in cervico-dorsal spine (C6-D4) with intramedullary bleed (white arrow).

**Figure:**
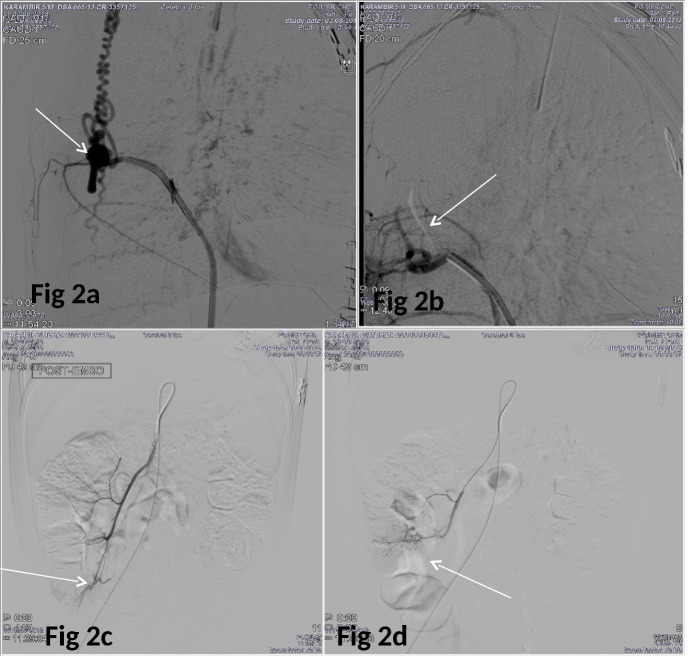
Figure 2: Digital subtraction angiography showing dural arteriovenous fistula (AVF) (arrow in 2a) with arterial feeder from radiculopial branches of right D6 intercostal artery Post embolization angiography showed glue cast at fistula site and no opacification of arterial feeder (arrow in 2b). Selective angiogram of feeding branch of superior mesenteric artery showing arteriovenous malformation in cecal region (arrow in 2c). Post-embolization selective angiogram shows complete obliteration of arteriovenous malformation (arrow in 2d) with patency of rest of the adjacent vessels.

## DISCUSSION

Spinal cord vascular malformation is a rare entity, and it accounts for 2–4% of spinal diseases.[3] Vascular malformations of the gastrointestinal tract are also rare in children.[2] Concurrent spinal and gut AVM is a rarity. Multiple AVMs are usually associated with hereditary disorders such as Rendu–Osler–Weber syndrome or hereditary hemorrhagic telangiectasia. It is extremely uncommon to find concurrent multiple AVM not associated with such syndromes. Multiple AVMs may be result of yet unrevealed pathogenesis or strong embryogenetic anomaly, which may be different from that involved in single AVM.[4]

AVMs arise because of failure of embryogenesis during the differentiation of vascular channels into mature arteries, capillaries, and veins, resulting in direct arteriovenous shunts without intervening capillary beds.[5] Spinal cord AVM are shunts, which are fed by arteries normally supplying the neural tissue, whereas spinal dural AVFs are fed by radiculo-meningeal arteries.[6] Screening for multiple AVMs is warranted in cases when a single lesion does not explain the presenting features.[7]

Common presentation of colonic vascular malformations in children are lower intestinal bleeding, intussusception, intestinal obstruction or intestinal perforation. The diagnosis is often delayed until severe anemia or chronic hemorrhage ensues. A negative initial workup should be followed by angiography to prevent delay in diagnosis and its complications.[8]

Main aim of treatment is complete angiographic obliteration of the AVM. Modalities like microsurgery, endovascular embolization, and stereotactic radiosurgery have an established role in the treatment of patients with AVM, and a staged approach has been proposed for patients with multiple AVMs.[9] The case deserves attention for variety of reasons including a rare association of spinal dural AVF and cecal arteriovenous malformation, its non-syndromic association and potential for cure.

## Footnotes

**Source of Support:** Nil

**Conflict of Interest:** None declared

